# The utility of DNA methylation signatures in directing genome sequencing workflow: Kabuki syndrome and CDK13‐related disorder

**DOI:** 10.1002/ajmg.a.62650

**Published:** 2022-01-18

**Authors:** Ashish Marwaha, Gregory Costain, Cheryl Cytrynbaum, Roberto Mendoza‐Londono, Lauren Chad, Zain Awamleh, Eric Chater‐Diehl, Sanaa Choufani, Rosanna Weksberg

**Affiliations:** ^1^ Department of Medical Genetics Cumming School of Medicine, The University of Calgary Calgary Alberta Canada; ^2^ Division of Clinical and Metabolic Genetics The Hospital for Sick Children Toronto Ontario Canada; ^3^ Genetics and Genome Biology Program Research Institute, The Hospital for Sick Children Toronto Ontario Canada; ^4^ Department of Pediatrics University of Toronto Toronto Ontario Canada; ^5^ Department of Molecular Genetics University of Toronto Toronto Ontario Canada; ^6^ Institute of Medical Sciences, University of Toronto Toronto Ontario Canada

**Keywords:** *CDK13*, DNA methylation signature, Kabuki syndrome, *KDM6A*, *KMT2D*

## Abstract

Kabuki syndrome (KS) is a neurodevelopmental disorder characterized by hypotonia, intellectual disability, skeletal anomalies, and postnatal growth restriction. The characteristic facial appearance is not pathognomonic for KS as several other conditions demonstrate overlapping features. For 20‐30% of children with a clinical diagnosis of KS, no causal variant is identified by conventional genetic testing of the two associated genes, *KMT2D* and *KDM6A*. Here, we describe two cases of suspected KS that met clinical diagnostic criteria and had a high gestalt match on the artificial intelligence platform Face2Gene. Although initial KS testing was negative, genome‐wide DNA methylation (DNAm) was instrumental in guiding genome sequencing workflow to establish definitive molecular diagnoses. In one case, a positive DNAm signature for *KMT2D* led to the identification of a cryptic variant in *KDM6A* by genome sequencing; for the other case, a DNAm signature different from KS led to the detection of another diagnosis in the KS differential, CDK13‐related disorder. This approach illustrates the clinical utility of DNAm signatures in the diagnostic workflow for the genome analyst or clinical geneticist—especially for disorders with overlapping clinical phenotypes.

## 1 INTRODUCTION

Kabuki syndrome (KS) was first reported by Japanese physicians in 1981 (Kuroki et al., 1981; Niikawa et al., 1981) and is now established as a recognizable syndrome with the cardinal features of facial dysmorphology, skeletal anomalies, dermatoglyphic abnormalities, intellectual disability, and postnatal growth retardation. In 2018, an international consensus was reached on diagnostic criteria (Adam et al., 2019). The authors proposed a clinical diagnosis be based on the presence of infantile hypotonia, developmental delay, and typical dysmorphic features (arched and broad eyebrows with lateral notching/sparseness, short columella with depressed nasal tip, large prominent or cupped ears, and persistent fingertip pads). Pathogenic variants in the genes *KMT2D* and *KDM6A* are known to be causal in Kabuki syndrome KS1 [MIM: 147920] and KS2 [MIM: 300867] cases, respectively (Banka et al., 2012; Lederer et al., 2012; Miyake et al., 2013; Ng et al., 2010). Among patients with a clinical diagnosis of KS, 75% are attributable to pathogenic variants in KMT2D and 3%–5% to pathogenic variants in KDM6A (Adam et al., 1993; Bogershausen et al., 2016). These two genes have opposite functions: *KMT2D* encodes a histone methyltransferase, whereas *KDM6A* encodes a lysine demethylase. The proteins encoded by these two genes form a functional complex that underpins the pathogenic mechanisms of both types of KS through the developmental epigenetic dysregulation of multiple genes. For 20%–30% of children, with a clinical diagnosis of KS, the genetic cause remains unknown (Adam et al., 1993; Bogershausen & Wollnik, 2013; Bogershausen et al., 2016). Reduced sensitivity of genetic testing could result from: locus heterogeneity due to unidentified novel genes; deep intronic variants beyond the detection limits of current gene panel testing (structural variants, promoter, or regulatory variants); or the existence of syndromes with overlapping phenotypic features.

Functional assays, such as genome‐wide DNA methylation (DNAm) analysis, can help clarify some of these diagnostic challenges. We and others have previously described a gene‐specific *KMT2D* DNAm “signature” defined as specific sites of differential DNAm in peripheral blood of individuals with pathogenic variants in *KMT2D* (Butcher et al., 2017). These signatures have been used to build models to classify variants of uncertain significance (VUS) in *KMT2D* as pathogenic (overlapping the KS DNAm profile) or benign (overlapping the control DNAm profile) (Aref‐Eshghi et al., 2017, 2019). We also found that individuals with KS due to a *KDM6A* pathogenic variant had a DNAm signature overlapping that of *KMT2D* (Butcher et al., 2017). This is not surprising as the proteins KDM6A and KMT2D form a functional complex. Overlapping DNAm signatures have previously been identified for genes encoding proteins that form complexes, for example, BAF (Aref‐Eshghi et al., 2018) and PRC2 (Choufani et al., 2020). Therefore, the *KMT2D* signature can be used to identify patients with KS secondary to pathogenic variants in the *KDM6A* gene (Sadikovic et al., 2021).

It is important to determine the exact genetic etiology in patients who meet the clinical diagnostic criteria for KS because the information informs recurrence risk, prenatal testing options, and potential gene‐targeted therapies (Zhang et al., 2021). Here, we describe two patients who were initially given a working clinical diagnosis of KS despite negative genetic testing (sequencing and deletion/duplication analysis of *KMT2D* and *KDM6A*). DNAm analysis demonstrated a positive KS signature in one patient congruent with the subsequent result on genome sequencing that identified a single exon duplication in the *KDM6A* gene, consistent with a diagnosis of KS. In the other patient, a DNAm signature different from *KMT2D* and controls, suggested the genomic alteration was not in either *KMT2D* or *KDM6A*. Genome sequencing identified a pathogenic variant in *CDK13*, which causes a syndrome with cardinal distinctive features overlapping KS called CDK13‐related disorder.

## 2 METHODS

### 2.1 DNA methylation array processing

Genome‐wide DNAm profiling was completed for typically developing controls (*n* = 45), the two patients, along with individuals with pathogenic *KMT2D* (*n* = 9) and *KDM6A* (*n* = 1) variants at The Center for Applied Genomics, SickKids Research Institute. Whole‐blood genomic DNA from each subject was sodium bisulfite converted using the EpiTect Bisulfite Kit (EpiTect PLUSBisulfite Kit, QIAGEN), according to the manufacturer's protocol. Modified genomic DNA was then processed and analyzed on the Infinium HumanMethylationEPIC BeadChip (Illumina 850K) according to the manufacturer's protocol. The raw IDAT files were converted into beta‐values, which represent DNAm levels as a percentage (between 0 and 1), using the minfi Bioconductor package in R as previously reported (Choufani et al., 2020). All samples passed standard quality control metrics in *minfi*.

### 2.2 Generation of machine learning scores for variant classification

Using our established DNAm signature for *KMT2D* and the Support Vector machine (SVM) model as previously described (Butcher et al., 2017; Turinsky et al., 2020) beta values were imported into EpigenCentral (https://epigen.ccm.sickkids.ca) (Turinsky et al., 2020) to impute SVM classification scores for all samples tested using the *KMT2D* SVM model. The model was set to the “probability” mode to generate SVM scores ranging between 0 and 1 (or 0% and 100%), thus classifying samples as “KS” (high scores) or “not‐KS” (low scores). This SVM model was built as a tool for the classification of variants in *KMT2D* and *KDM6A* as previously described (Butcher et al., 2017).

### 2.3 Genome sequencing

Genome sequencing was performed at the Centre for Applied Genomics with high‐quality DNA extracted from whole blood using established methods (Costain et al., 2020; Lionel et al., 2018). Patient 1 was included as individual “CMC 16” in a previous cohort study (Costain et al., 2020). Sequence data were analyzed to identify putative disease‐associated variants as previously described (Costain et al., 2020; Lionel et al., 2018). Variants were confirmed by an orthogonal method in a CLIA/CAP approved clinical laboratory and returned to the families accompanied by genetic counseling.

### 2.4 Consent

Informed consent was obtained from all research participants according to the protocol approved by the Research Ethics Board of the Hospital for Sick Children (REB #1000038847). Separate consent was obtained to use a 2‐dimensional facial photograph in the Face2Gene platform and to perform DNAm analysis (REB #1000066122).

## 3 PATIENTS

### 3.1 Patient 1

**FIGURE 1:**
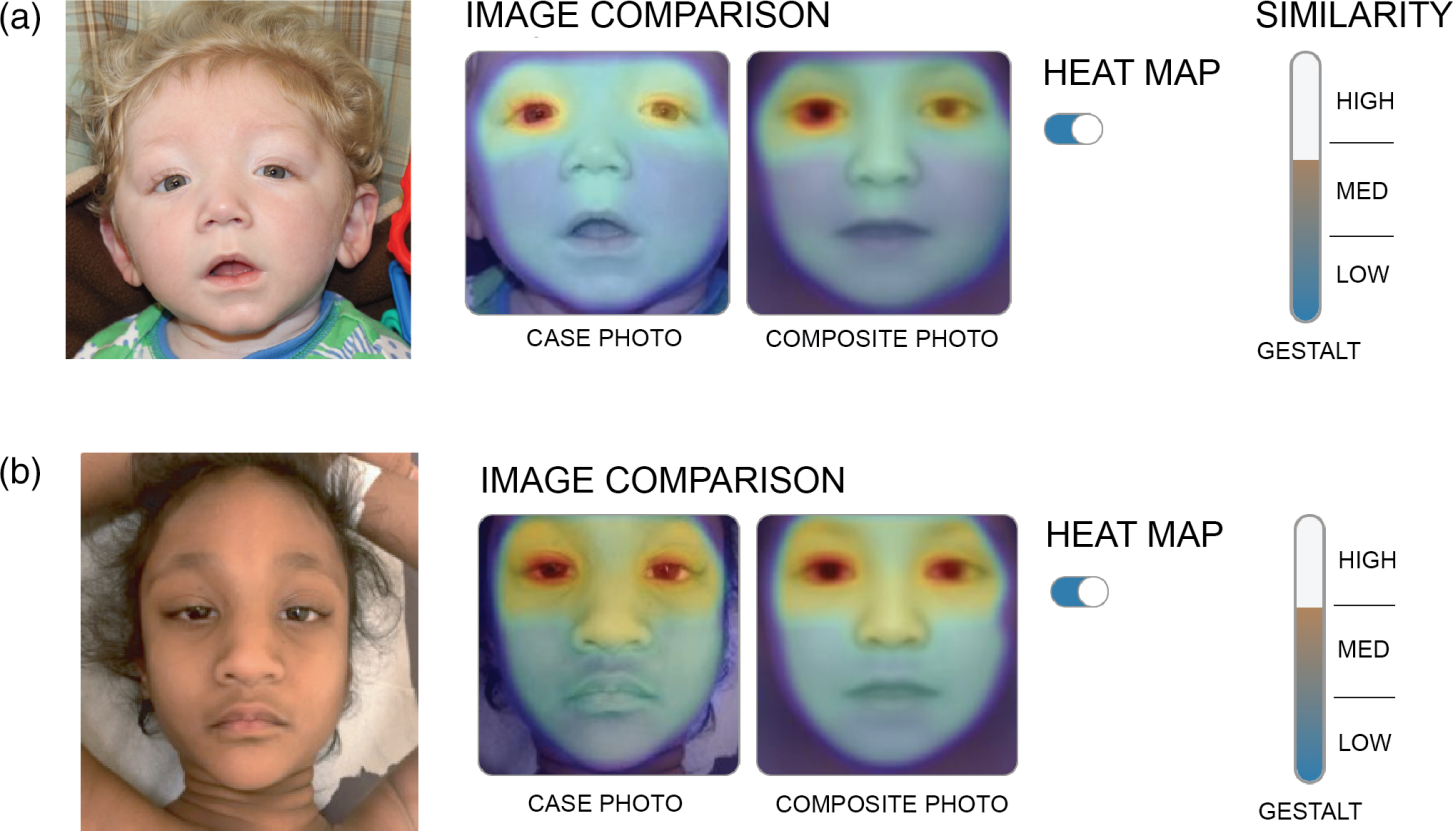
Facial gestalt of patient 1 (a) and patient 2 (b). Facial raw images are shown aligned to the composite image produced by the Face2Gene software for Kabuki syndrome (KS). Similarity scale reflective of the gestalt score for a match to KS is also shown

A 9‐year‐old boy of British and Bulgarian ancestry, born to non‐consanguineous parents was first seen in the neonatal period for a genetics consult when he presented with congenital hyperinsulinism. His subsequent medical issues included global developmental delay, postnatal growth retardation, hypotonia in infancy, cortical visual impairment, seizures, and severe gastrointestinal reflux disease requiring G‐tube feeds. Magnetic resonsance imaging (MRI) of the brain showed remote bilateral matrix hemorrhages, but no structural malformations. He had renal cysts detected on ultrasound and a normal echocardiogram. His clinical examination at 10 months of age was in keeping with KS and in addition to his characteristic facial features (Figure 1a), he had a high arched palate, pectus excavatum, and scoliosis. He met criteria to receive a clinical working diagnosis of KS (Adam et al., 2019).

**FIGURE 2:**
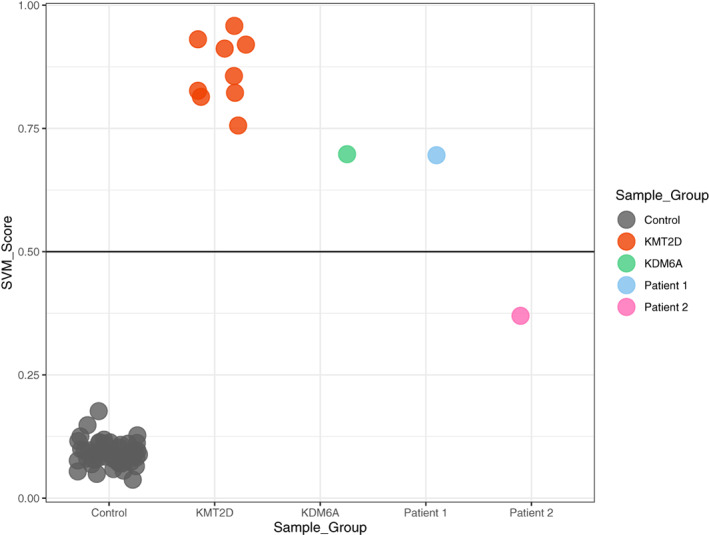
DNA methylation analysis for patients. Plot representing the Support Vector machine (SVM) scores (*y* axis). The SVM prediction model was used to predict pathogenicity of variants based on the *KMT2D* DNA methylation signature. All nine *KMT2D* variants were classified as pathogenic. All 45 control samples had very low SVM scores as expected. The blue dot represents patient 1 (single‐exon duplication in *KDM6A*), which had a high SVM score and therefore classified as pathogenic for Kabuki syndrome (KS) similarly to a previously published KS case with pathogenic *KDM6A* variant (green dot, Butcher et al., 2017). The pink dot represents patient 2 with a *CDK13* pathogenic variant (c.21149G>A) and shows this individual had an intermediate score using the *KMT2D* signature

We performed genetic testing over many years using a tiered approach. Chromosome microarray analysis, targeted *KDM6A* and *KMT2D* gene sequencing and multiplex ligation‐dependent probe amplification (MLPA) analysis and exome sequencing were all non‐diagnostic. DNAm analysis showed that this patient clearly classified as positive with the *KMT2D* signature (Figure 2) and had a SVM score of 0.7, that is, his DNAm profile clustered with the DNAm signature identified for patients with a genetically confirmed diagnosis of KS.

**FIGURE 3:**
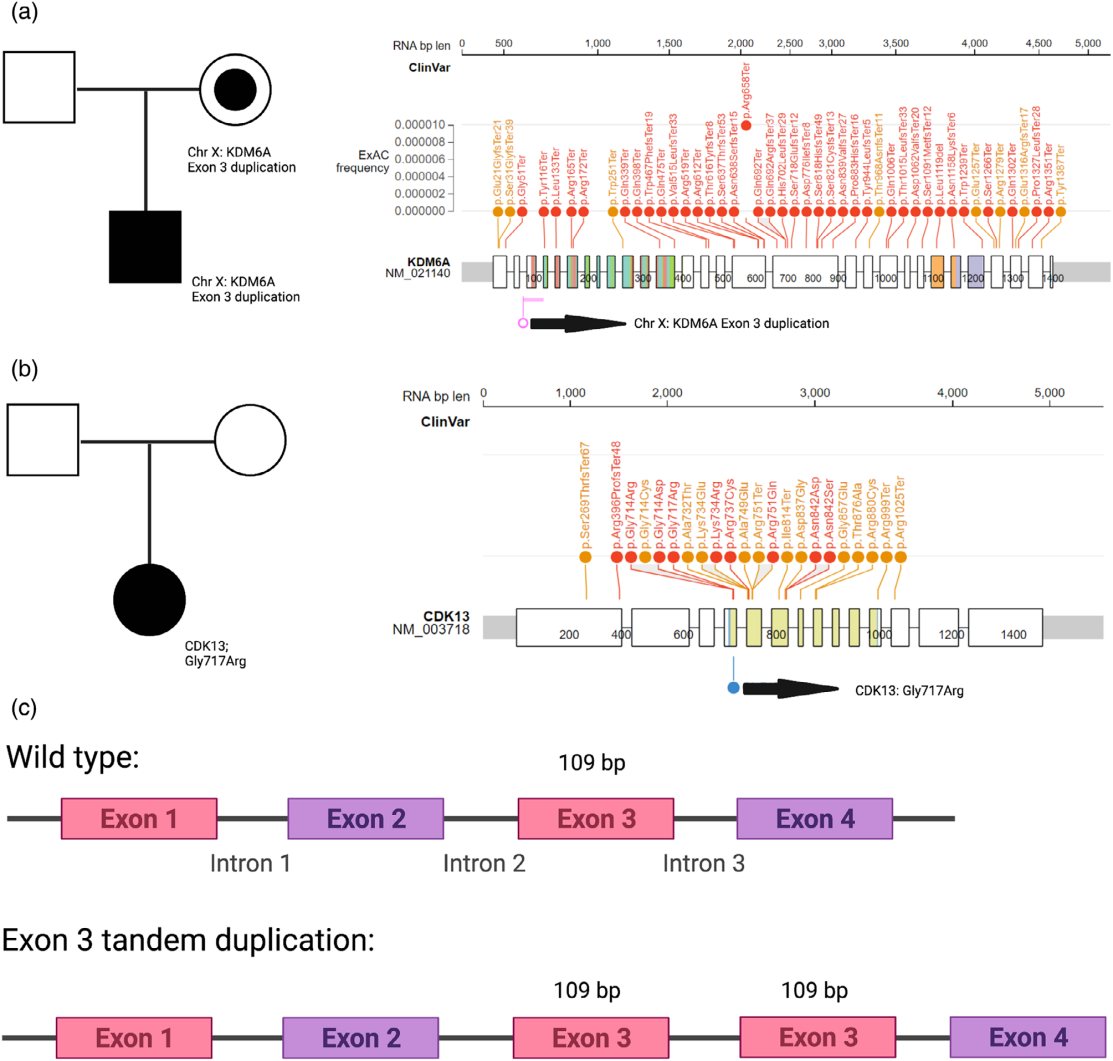
Pedigrees and variant description for patients. Pedigrees and variant location in the gene are show for patient 1 (a) and patient 2 (b). The location of other known pathogenic variants in the *KDM6A* and *CDK13* genes are also shown for reference. Panel (c) describes how the exon 3 tandem duplication in patient 1 results in a 109 bp insertion, which would be predicted to cause a frameshift

We performed trio genome sequencing, which identified an ~8 kb duplication encompassing exon 3 in *KDM6A* predicted to result in a frameshift due to a 109 bp insertion as shown in Figure 3 (NM_001291421.2:g.44818001_44826000dup) (Costain et al., 2020). This small copy number variation was not detected by chromosomal microarray analysis, targeted gene testing (including sequencing and MLPA), or clinical exome sequencing. Calling single‐exon level copy number variants (CNVs) by exome sequencing remains technically challenging, especially for duplications. In this case, exome sequencing was performed in a large, experienced CLIA/CAP‐approved laboratory. Data published by this laboratory indicate incomplete sensitivity for detection of clinically significant CNVs by exome sequencing (Dharmadhikari et al., 2019; Gambin et al., 2017). We would have expected the duplication to be detected by MLPA. After informing the original testing lab of the duplication detected on genome sequencing, they reviewed their results and told us that the exon 3 probe was “top normal” (just less than their cut‐off point for calling a duplication). The testing lab subsequently re‐ran the MLPA using a new kit which did detect the duplication.

The variant was determined to be maternally inherited and therefore had important implications for future pregnancy planning (Figure 3). The facial recognition platform Face2Gene was used retrospectively to provide a list of syndromic matches based on his picture at 10 months of age (Gurovich et al., 2019; Marwaha et al., 2021). A gestalt score over 0.5 is highly indicative of a genuine phenotype match (Marwaha et al., 2021). Face2Gene analysis placed KS as the top match with a gestalt score of 0.61.

### 3.2 Patient 2

A 9‐year‐old girl of Indian descent, born to non‐consanguineous parents, was referred for a genetics assessment due to a history of severe developmental delay (non‐verbal), microcephaly, hypotonia, severe feeding issues in infancy, postnatal growth retardation, and esotropia. A brain MRI showed no structural abnormalities. She had a normal renal ultrasound and echocardiogram. On clinical examination, she was noted to have dysmorphic features consistent with KS, including high arched eyebrows and eversion of the lower eyelids (Figure 1b) and fetal fingertip pads. The top match on Face2Gene was KS with a gestalt score of 0.66 (range 0–1). She also met criteria to receive a clinical working diagnosis of KS (Adam et al., 2019).

We performed targeted genetic testing (sequencing and deletion/duplication analysis of the *KDM6A* and *KMT2D* genes) via a commercial platform, which did not identify any pathogenic variants. Since we were still highly suspicious of a diagnosis of KS, we performed DNAm analysis. Unexpectedly, she had a unique DNAm profile that did not cluster with either typically developing controls or with individuals with KS and pathogenic *KMT2D* or *KDM6A* variants ‐ an intermediate SVM score (SVM score = 0.4) (Figure 2) was obtained. In studies of other disorders, we have found that intermediate scores often represent specific phenomena such as somatic mosaicism, atypical variants in the same gene, or variants in related genes (Chater‐Diehl et al., 2019; Goodman et al., 2020). We performed trio genome sequencing, which showed she had a de novo missense variant in the *CDK13* gene as shown in Figure 3 (NM_003718.5): c.2149G>A; p.(Gly717Arg). The variant is absent in large‐scale population databases of genomic variation (gnomAD and TOPMed), is predicted to be damaging by in silico tools, and has been previously classified in multiple individuals as pathogenic (ClinVar Accession: VCV000375737.7). *CDK13*‐related disorder (MIM #617360)—also known as congenital heart defects, dysmorphic facial features, and intellectual developmental disorder—has previously been noted to present with a KS‐like phenotype (Bostwick, 1993). No other candidate variants were identified by genome sequencing, including in *KMT2D* and *KDM6A*.

## 4 CONCLUSION

Despite the development of International Consensus criteria for the diagnosis of KS, it is useful to have alternative avenues to identify a molecular diagnosis if testing does not confirm a pathogenic variant in *KMT2D* or *KDM6A*. A functional test such as genome‐wide DNAm can be a useful adjunct to defining an accurate diagnosis. Here, we present two patients that illustrate how this approach can either reliably confirm that further assessment of a candidate gene is indicated or that an alternative diagnosis is more likely, in which case further testing using exome or genome sequencing should focus on potential pathogenic variants in other genes, in particular those that can be associated with overlapping phenotypes, for example, CDK13‐related disorder. For our patient with CDK13‐related disorder, although the variant would have been detected on exome sequencing, we opted for genome sequencing as this was available to us through a research study and provided the more comprehensive testing option. Genome sequencing has the added benefit that analytical detection of CNVs is at least equivalent to chromosomal microarray analysis, which is not the case for exome sequencing (Marshall et al., 2020). The recently described CDK13‐related disorder phenotype indicates that most patients have overlapping cardinal features of KS (Bostwick, 1993; Hamilton & Suri, 2019). Most experts in dysmorphology would have difficulty differentiating the two conditions based on facial appearance alone. Currently, facial recognition software such as Face2Gene also cannot accurately discriminate between the two conditions, but the software could likely be trained to identify a gestalt for CDK13‐related disorder if more cases are uploaded. The pathogenesis of CDK13‐related disorder has not been completely elucidated though we know that cyclin dependent kinases are serine threonine kinases that can regulate gene transcription by phosphorylation of serine residues (Hamilton & Suri, 2019). Since we show in patient 2 that a *CDK13* variant impacts genome‐wide DNAm, the mechanisms of disease likely include features of epigenetic dysregulation. These data also suggest the existence of a gene‐specific *CDK13* signature which is under investigation. Although CDK13‐related disorders have a similar facial gestalt to Kabuki, the distinctness of the genomic targets identified by the methylation pattern in Patient 2, indicate that the former has a separate pathogenic mechanism. This suggests that CDK13‐related disorder is likely to be a distinct clinical syndrome as opposed to “Kabuki syndrome 3.” As we learn more about the basic biology of conditions with overlapping phenotypes, the inclusion of both genotype and phenotype data into nomenclature, as recently proposed by Biesecker et al. (2021) will allow for more accurate definition of complex syndromic disorders.

We suggest that in cases of suspected KS with negative targeted panel testing, utilization of genome‐wide DNAm and consideration of facial phenotyping tools could help provide direction for further genetic testing to improve the efficiency of the diagnostic workflow. DNAm can be used to streamline further genetic testing—in some cases suggesting more detailed analysis of a specific gene and in other cases expanding to genome‐wide sequencing, which is more costly and not universally available in the clinical setting. The utility of DNAm profiling in classifying VUS , arising from genomic sequencing, has already been demonstrated for a large number of rare neurodevelopmental disorders caused by pathogenic variants in genes that affect epigenetic regulation (Choufani et al., 2020; Cytrynbaum et al., 2019; Rots et al., 2021; Sadikovic et al., 2021). Recent work has even suggested that commercially available methylation analysis can be used as a first line diagnostic test for neurodevelopmental conditions (Sadikovic et al., 2021). Once a DNAm profile is available for a patient sample, it can be compared bioinformatically to all available DNAm signatures, that is, currently greater than 40 signatures including *KMT2D*. At this time, there is not a DNAm signature for *CDK13*, although we anticipate in the future this signature will be defined. However, not all genes will be associated with a DNAm signature. If DNAm is not diagnostic, then reflexing to genomic sequencing would be the next logical step. A positive DNAm classification can establish a diagnosis, but additional testing would be required to identify the pathogenic sequence variant. Since DNAm analysis does not identify the causal variant, it cannot be a stand‐alone first‐line test. DNAm testing also has important limitations, which must be considered when the testing is utilized in a clinical context but might not be widely understood. The DNAm signatures developed are only validated for peripheral blood samples and may not apply to other tissue types. Sample size used for generating the signature, age at the time of blood sampling and other external factors can lead to reduced reproducibility in reliably using a specific signature for diagnosis (Chater‐Diehl et al., 2021). Our two patients illustrate the use of DNAm analysis in the diagnostic workflow of the common clinical presentation of suspected KS. We propose that DNAm analysis is best used in conjunction with other evidence (facial analysis and genomic sequencing) to help classify patients with diagnostic uncertainty after first line clinical molecular testing.

## CONFLICT OF INTEREST

The authors have no conflict of interests to declare.

## AUTHOR CONTRIBUTIONS

Ashish Marwaha is the primary author of the manuscript, gathered clinical report data and helped direct data analysis. Gregory Costain performed the whole genome sequencing analysis and reviewed the manuscript. Cheryl Cytrynbaum and Roberto Mendoza‐Londono contributed to the clinical data reported and reviewed the manuscript. Lauren Chad reviewed the manuscript. Zain Awamleh, Eric Chater‐Diehl, and Sanaa Choufani performed methylation sequencing and analysis and reviewed the manuscript. Rosanna Weksberg secured ethics approval and funding for the data collection and analysis, she also co‐wrote the manuscript, directed data analysis/presentation and is corresponding author.

## Data Availability

The data that support the findings of this study are available on request from the corresponding author. The data are not publicly available due to privacy or ethical restrictions.
